# TREM-1; Is It a Pivotal Target for Cardiovascular Diseases?

**DOI:** 10.3390/jcdd5030045

**Published:** 2018-09-07

**Authors:** Kouassi T. Kouassi, Palanikumar Gunasekar, Devendra K. Agrawal, Gopal P. Jadhav

**Affiliations:** Department of Clinical and Translational Sciences, School of Medicine, Creighton University, Omaha, NE 68178, USA; TataKouassi@creighton.edu (K.T.K.); KumarGunasekar@creighton.edu (P.G.); DKAgr@creighton.edu (D.K.A.)

**Keywords:** triggering receptor expressed on myeloid cells, DNAX-activating protein 12, cardiovascular diseases, atherosclerosis, coronary arteries diseases, acute myocardial infract, endocarditis

## Abstract

Cardiovascular diseases (CVDs) are as menacing as ever and still continue to kill adults worldwide, notwithstanding tremendous efforts to decrease their consequent mortality and morbidity. Lately, a growing body of research indicated that inflammation plays a pivotal role in the pathogenesis and complications of CVDs. A receptor of the immunoglobulin superfamily, triggering receptors expressed on myeloid cells-1 (TREM-1) was shown to induce and amplify the inflammation in both acute and chronic disease’ pathogenesis and progression, which hence makes it one of the most important complication factors of CVDs. Thus, studies endeavored to investigate the role played by TREM-1 in CVDs with respect to their etiologies, complications, and possible therapeutics. We examined here, for the first time, the most relevant studies regarding TREM-1 involvement in CVDs. We critically analyzed and summarized our findings and made some suggestions for furtherance of the investigations with the aim to utilize TREM-1 and its pathways for diagnostic, management, and prognosis of CVDs. Overall, TREM-1 was found to be involved in the pathogenesis of acute and chronic cardiovascular conditions, such as acute myocardial infarction (AMI) and atherosclerosis. Although most therapeutic approaches are yet to be elucidated, our present research outcome displays a promising future to utilizing the TREM-1 pathway as a potential target for understanding and managing CVDs.

## 1. Introduction

Cardiovascular diseases (CVDs) still hold the first place in causing death in adults in most parts of the world, despite the ongoing battle against them [1,2,3]. It is important to know that the fight against CVDs is weakened by the continuous increase in the prevalence of metabolic syndrome, which is their bedrock underlying etiology [2]. Conditions such as sedentariness, high cholesterol, and high-fructose diets also exact a heavy toll on CVDs’ prevalence. The common denominator of the onset and progression of these diseases is the chronic immunoinflammatory process that involves the upregulation of proinflammatory cytokines and chemokines, such as IL-1, IL6, TNF-α, and IL-8 [4,5,6]. TREM-1 has been found to be involved in acute [7,8] and chronic inflammatory diseases, owing to its role played in innate immunological reactions [9,10,11]. Lately, investigations have endeavored to find the implications of TREM-1 in the pathogenesis and management of CVDs. Overall, the findings are promising for the TREM-1 pathway to be utilized as CVD biomarkers or for their management [12,13,14,15].

TREM-1 is a 30 kD glycoprotein surface receptor that belongs to the superfamily of immunoglobulin [16,17]. It is initially found on cells derived from myeloblast, where it amplifies their inflammatory responses with the release of cytokines and chemokine [18]. Bacterial and fungal infections stimulate TREM-1 expression [8,19]. There are two relevant forms of TREM-1: the trans-membrane, the soluble -or the secreted (sTREM-1). While the overall expression of all TREM-1 is associated with an increased severity of infection, the transmembrane form is found to contribute to the inflammation process while the soluble form plays a role of decoy receptor in amending the inflammatory process [20]. In fact, sTREM-1 hinders the TREM-1 ligand to reach the monocytes, macrophages, and neutrophils membrane [21,22]. It has been found that sTREM is released by metalloproteinase activity which detaches the ectodomain of the transmembrane TREM-1 [23].

We review, in this article, the findings of recent publications regarding the implications of TREM-1, mainly in heart- and vessel-related diseases. We will assess the strength of the results and present the relationships between them, and will also suggest new pathways of investigation that will corroborate with the current findings to advancing the therapeutics of CVDs, utilizing the TREM-1 pathway.

## 2. TREM-1 Signaling

**Figure 1:**
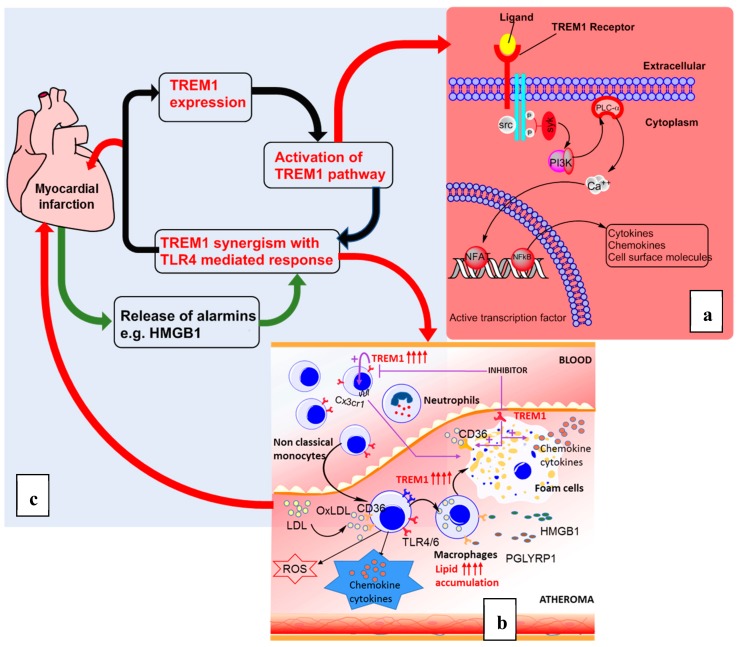
Triggering receptors expressed on myeloid cells-1 (TREM-1) in the pathogenesis of atherosclerosis leading to acute myocardial infarction. (**a**) oxidized low density lipoprotein (oxLDL) concentration elevation in the plasma induces TREM-1 upregulation and its ligand interaction, leading to downstream signaling. TREM-1 interacts with DAP-12 to induce proinflammatory cytokines, chemokines, and cell surface molecule transcription through PI3K and PLC-α and nuclear factors NFAT and NF-κB. (**b**) The chronic inflammation condition induces the proliferation, recruitment, and differentiation of monocytes to macrophages which, in turn, uptake the oxLDL to form foam cells. It also induces the proliferation of smooth muscle cells (SMCs) that play a similar role. Altogether they form the atheroma. (**c**) The destabilization of the atheroma plaque causes coronary artery clog, resulting in acute myocardial infarction (AMI). During AMI, TREM-1 upregulation also induces a deleterious cardiac wall scar, which is a source of heart failure.

The membrane-bound TREM-1 has a short intracellular domain. It requires association with the immunoreceptor tyrosine-based activation motif (ITAM), containing a signaling adaptor protein, a DNAX-activating protein 12 (DAP-12), as well as interaction with MyD88 to carry out its intracellular signaling (Figure 1a). It is established that TREM-1 and toll-like receptors (TLRs) interact synergistically to promote inflammation by inducing the production of inflammatory cytokines and chemokines. TLRs are the most preeminent pathogen recognition receptors (PRRs) that carry out inflammatory reactions upon stimulation by pathogen-associated molecular patterns (PAMPS) or damage-associated molecular patterns (DAMPs) [22,24,25,26,27]. In fact, TREM-1 interacts with TLR-4 to induce more cytokine production by myeloid cells upon exposure to LPS [28,29]. The process involves the recruitment of inflammation mediators such as Phosphatidylinositol-4,5 bisphosphate 3-kinase (PI3K), extracellular signal–regulated kinases (ERKs), interleukin-1 receptor-associated kinase 1 (IRAK-1), and nuclear factor kappa-light-chain-enhancer of activated B cells (NF-κB) [30]. Moreover, it has been found that the activation of TREM-1 alone does not induce a consistent inflammation process. This suggests that TREM-1 relies on its interaction with other pathways, such as of TLRs, to fully carry out its downstream reactions, as far as inflammation is concerned. In the cytoplasm, nucleotide-binding oligomerization domain (NOD)-like receptors (NLRs) play a similar synergistic role with TREM-1 [31]. As PRRs, they recognize both PAMPs and DAMPs in microbial infections, as well as sterile tissue injuries. Upon stimulation, they induce the recruitment of innate immune cells that in turn initiate the adaptive immune response. NOD-1/2 activation, in conjunction with TLRs and TREM-1, induce NF-κB upregulation and other pro-inflammatory cytokines. It is yet to be elucidated whether NLRs and TREM-1 can react synergistically but independently from TLRs.

### TREM-1 Ligands

Earlier on in this decade, putative ligands of TREM-1 used to be evasive [29,32], but lately, light started to shine on them. High-mobility group box-1 (HMGB-1), also known as amphoterin, was the earlier targeted ligand for its role in inducing TREM-1 signaling in the activation of the Kupffer cell in hepatocellular carcinomas [33,34,35,36]. It plays a very significant role as chromatin proteins that, like histones, are involved in DNA stabilization and gene transcription control. It is constantly upregulated in macrophages and neutrophils which are recruited during the inflammation process, especially in tissue injury [37,38]. HMGB-1 also activates PRRs such as DAMPs. It is found to upregulate NF-kB, and translocates to the nucleus for pro-inflammatory cytokine and chemokine production. Also, in inflammation, HMGB-1 works in concert with receptors of advanced glycation end products (RAGE) or TLRs, to form a complex with CXCL-12 (HMGB-1-CXCL-12) in synergy with the stromal cell-derived factor-1 (SDF-1) to enhance CXCR-4 signaling and inflammatory cell migration [39].

Peptidoglycan recognition protein-1 (PGRP-1), by itself or linked to its ligand-peptidoglycans (PG) [40], was found to induce TREM-1 signaling and synergetic interaction with TLRs [41,42]. PGs are glycopeptide polymers containing *N*-acetylmuramic acid or *N*-glycolylmuramic acid and d-amino acids. They are found in the bacterial wall, where they play a key role in maintaining bacterial morphology. Unlike endotoxins, which are found exclusively in gram-negative bacteria walls, PG is found in both gram-negative and gram-positive bacterial walls. Contrariwise, eukaryotic cells do not contain PG in their cell walls. PGRPs are recognized as types of PRR. They are actively involved in the innate immune response system, including pathogen recognition, phagocytosis induction, and degradation of the amidase activity of PG [43,44,45].

Actin was also found to trigger TREM-1 with direct interaction with the extracellular portion of the receptor and to induce downstream signaling in platelets and macrophages. Briefly, TREM-1 interaction with its ligand also induces a series of production of intermediary reactions, ultimately leading to the upregulation of pro-inflammatory cytokines, such as IL-1β, IL-2, IL-12, and TNF-α [46].

## 3. TREM-1 in Inflammatory Processes

TREM-1 upregulates the cell surface activation markers in myeloid cells, which also increases during septic shock. It is over-expressed in a number of infectious diseases, such as pneumonia and suppurated cholangitis [47,48,49]. Lately, several studies showed that TREM-1 upregulation in parenchymal cells is associated with the development of chronic disease in the organs they are found in [10,50,51,52]. In fact, TREM-1 was found to be upregulated in obstructive nephropathies [53] and chronic kidney diseases [9,54]. TREM-1 has been found to be constantly upregulated in chronic inflammatory bowel disease, especially during flare-up episodes [55]. TREM-1 has also been found to be implicated in glenohumeral arthritis development and its progression [56]. Macrophages in lung cancers were shown to increase TREM-1 expression with subsequent inflammatory responses, ultimately inducing complications and early death [8].

## 4. TREM-1 in Cardiovascular Diseases

Of the most recent studies from Medline with key words such as TREM-1, cardiovascular diseases, myocardial infarcts, endocarditis, atherosclerosis, and pericarditis, the most relevant studies we found are summarized in Table 1. It is remarkable how all of them approach cardiovascular diseases involving TREM-1 and the role it plays in inflammation either in acute settings like endocarditis and AMI, or in chronic conditions, such as in atherosclerosis (Table 1). In atherosclerosis, the role of TREM-1 is found to be in chronic inflammation, leading to macrophages, leukocyte apoptosis, and necrosis, which contribute to fatty streak buildups [57,58]. It has also been found to induce plaque vulnerability by TREM-1 expression of the vascular smooth muscle cells (VSMCs) and dendritic cells [59,60].

The destabilization of the plaque leads to artery occlusion, and when it occurs in the coronary artery, it causes acute myocardial infarction (AMI). TREM-1 was also found to amplify the inflammation following myocardial infarction, and its modulation provides a better outcome for post-AMI in both the short and long term [15]. Studies which endeavored to find any polymorphisms in TREM-1 gene expression associated with atherosclerosis remain elusive in regard to their overall conclusion—however, it has been found that sTREM-1 increases when AMI is associated with single nucleotide polymorphisms (SNPs) [61,62]). Elevated levels of serum sTREM-1 is associated with stent-restenosis (ISR), and TREM-1 expression is also seen more in the VSMCs of the neointimal and medial regions of the stenotic artery. It is possible that TREM-1 inhibition could play a modulating role in this phenomenon [63,64]. In endocarditis, several TREM-1 SNPs [54] have been reported to be associated with endocarditis susceptibility in Caucasians, but it does not have any effect on the outcome of endocarditis. In heart surgery, TREM-1 expression is found to be associated with post-operative inflammation, and for heart transplant rejection, TREM-1 has been found to play a key role in CD4+ lymphocytes’ recruitment [65].

### 4.1. TREM-1 in Pathogenesis of Atherosclerosis

Atherosclerotic plaque constitutes the hallmark etiology of cardiac, cerebrovascular, and other embolismic events in individuals with metabolic syndrome and genetic abnormalities, as well as those who smoke, who are male, or are in old age. Atherosclerotic plaque occurs by an increase in low-density lipoprotein (LDL) concentration in the blood and is also possibly triggered by viral or bacterial infection [74,75,76]. Its pathogenesis is multifactorial, and it develops through a progressive chronic inflammatory process over decades. The inflammation starts in the endothelial cell wall. Once in contact with the vessel wall, LDL particles trigger a proliferation and migration of myeloid cells, such as neutrophil and monocyte/macrophages, toward the lumen. The innate immune response has been found to induce myeloid cell proliferation, which contributes to the formation of the plaque. In the beginning of plaque formation, there is a flux of blood neutrophil and maturation of resident monocytes into macrophages in the media and endothelium of cholesterol-rich areas of the vessel. Their scavenger receptors recognize how the oxidized low-density lipoprotein (oxLDL) engulfs and digests them. The excess accumulation of lipid turns them into cells occupied with large droplets of fat, called foam cells [5]. They contribute and maintain an environment of chronic inflammation with the release of cytokines and chemokines. If there is no reversion of the condition, leading to this initial chronic inflammation stage, the recruitment of inflammatory cells will continue and thus produce more foam cells, which will later undergo apoptosis and calcification [77]. This process will continue to build up the lipid streak and calcified plaque. If the endothelium is intact, the plaque remains stable. Destabilization is found to be a normal complication of growing plaques, especially vulnerable ones [67]. The vulnerable plaque has been found to cause diseases such as myocardial infarcts if they are in coronary arteries, or cerebrovascular stroke if they are in carotid arteries.

TREM-1 has been found to be upregulated in atherosclerotic plaque, compared to fibrotic plaque. Moreover, TREM-1 was found to promote the destabilization of atheroscelrotic plaque in association with an upregulation of matrix metalloproteinase (MMP) [78]. In a study comparing symptomatic and asymptomatic plaques, it has been found that TREM-1 and DAP-12 are upregulated in symptomatic plaques, compared to asymptomatic. Furthermore, the stimulation of VSMCs derived from symptomatic plaques, with the potent inflammatory cytokine TNF-α, induced a higher expression of TREM-1 compared to the ones from asymptomatic plaques. This suggests that VSMCs from unstable plaque are prone to inflammation, and TREM-1 plays a major role in this pathogenesis. The upregulation of TREM-1 in atherosclerotic plaque contributes to plaque destabilization via its upregulation in macrophages and VSMCs by inducing the secretion of MMP-1 and MMP-9, which hydrolyzes the gelatin and collagen and leads to the plaque’s vulnerability [67]. Interestingly, it has recently been found that MMP-9 has a pivotal role in cleaving the membrane-bound, TREM-1-inducing, increased circulation of sTREM-1 above the threshold, so that it is used as important biomarker in sepsis [20]. This finding suggests that TREM-1 and MMP-9 are positively correlated, and needs more elucidation. However, these studies implicate the significant role of this tandem in the pathogenesis of atherosclerosis. Moreover, l-selectin, vascular cell adhesion molecule-1 (VCAM-1), and intercellular adhesion molecule-1 (ICAM-1) were found to be key players in the building up of the atheroma plaque [68,79], which areupregulated by the increased expression of TNF-α, IL-4.

One of the dilemmas to overcome in the treatment of arteriosclerotic caused diseases is the restenosis of in-stent implants. In fact, in-stent restenosis [64] significantly restricts the hope offered by peripheral angioplasty after artery occlusion (Table 1). Several drugs have been suggested to reduce the chance of any demoralizing events happening, without any significant success. A recruitment of monocytes and low-grade inflammation has been found to play a key role in the proliferation of VSMCs [80]. sTREM-1 is shown to increase in patients with in-stent restenosis (ISR), and VSMCs isolated from the neo-intima show increased TREM-1 compared to control [63,64].

### 4.2. TREM-1 in Coronary Artery Disease (CAD) and Acute Myocardial Infarction (AMI)

Coronary artery disease and acute myocardial infarction are the most common reasons for medical emergency admission in the U.S. In fact, AMI occurs in the U.S. every 40 s. Every year, 525,000 new cases of AMI occur while 210,000 happen in people who have already had a heart attack. AMI occurs when a coronary artery is significantly occluded and unable to supply sufficient oxygenated blood to the area of the heart it irrigates. It is caused by indwelling atheroma plaque overgrowth, rupture, or by severe sudden vasoconstriction. This leads to occlusion by blood clot where platelets’ activation and aggregation is the key role player. The hypoxia resulting from this event induces myocardial tissue damage with cardiomyocyte necrosis and apoptosis. The release of DAMPS from this event has been found to trigger an inflammatory cascade that studies have found TREM-1 to be involved in (Figure 1) [14,69]. After the myocardial event, there is a release of inflammatory cytokine and chemokines which causes an influx of myeloid cells, such as monocytes and neutrophils, to the ischemic lesion area. They contribute to the normal myocardial repair with a fibrotic tissue [14,15]. Excessive upregulation of TREM-1 may induce, after long period of time, deleterious cardiac wall remodeling which impairs the cardiac morphology and function. Cardiac failure will occur with reduced heart output and contractility. Cardiac arrhythmia may also follow when the cardiac septum is affected [81,82].

TREM-1 is found to be implicated in AMI, where to the ischemic area, it recruits monocytes that differentiates into macrophages. It also causes neutrophil proliferation and their migration, like it does in bacterial lung infection [83]. Soluble TREM-1 is increased during the onset of MI and is a negative outcome predictor. Its increase correlates positively with an outcome of immediate overall death. It has also been found to determine late-onset heart failure following ST-segment elevation myocardial infarction (STEMI) in humans [15]. In mice, the inhibition of TREM-1 by LR-12 reduces monocyte and neutrophil recruitment, as well as inflammation in the affected myocardium. Also, inhibition of TREM-1 in its downstream signaling protects against heart failure, post-AMI. sTREM-1 is elevated in the blood during AMI, and its level is correlated with the outcome of death. Altogether, TREM-1 upregulation suggests complicated AMI, and early inhibition protects against detrimental outcomes [14,15,70]. Healing of the myocardial infract majorly involves leucocyte recruitment, which can cause damage if it not well-modulated. TREM-1 inhibition has been shown to eliminate neutrophil migration to ischemic sites, thus avoiding uncontrolled healing into deleterious scars. TREM-1 suppression should be explored as a preventive approach for AMI and for the management of post-AMI.

### 4.3. TREM-1 in Endocarditis

Endocarditis is the inflammation of the inner layer of the heart, and most commonly involves the cardiac valves. Its etiology is usually non-infective with chronic progression. This form is called nonbacterial thrombotic endocarditis (NBTE) because it does not involve bacterial infection in its development, and it clinically manifests by the release of the thrombus proceeding from the vegetation’s breakout. NBTE does not induce on its own a general inflammatory process, but only when complicated by bacterial infection, or by thrombotic events in the heart or brain. At times, further complications can also occur by bacterial infestation, causing infective endocarditis (IE) [84].

Acute-onset infective endocarditis is a life-threatening condition, and can be a very debilitating cardiovascular condition in the long run. TREM-1 was found to predispose Caucasian individuals to infective endocarditis. In fact, TREM-1 and its interaction with TLRs were consistently involved in acute infective diseases, such as pneumonia, septicemia, and gingivitis. Also, the blockage of TREM-1 in this condition has shown to decrease immune inflammatory reactions. In endocarditis among TLRs, rs3775073 polymorphism within the TLR-6 gene is the only one actually reported to be associated with the decrease of endocarditis onset among Caucasian peoples [54,74]. The application of this finding may lead therapeutic approaches to amend remodeling scars which follow the IE. Sepsis and hypercoagulation are the most common causes of NBTE. It is well-known that membrane-bound TREM-1 plays an important role in implications of immune response during sepsis, and there is also an increase of sTREM-1 in blood during sepsis.

## 5. Therapeutics of Cardiovascular Diseases Targeting TREM1 Pathway

To our knowledge, there has not yet been a specific therapeutic approach developed which targets TREM-1 pathways. Therapeutic approaches which are suggested by most studies are yet to be endeavored. Indeed, TREM-1 knockout mice have shown reduced inflammatory reactions when they were exposed to LPS or other inflammatory agents such PG, in acute as well in chronic disease settings. Trem-1^−/−^ mice were found to be resistant to severe forms of pneumonia, peritonitis, or septic shock [19,72,85]. The resistance that they developed is associated with the down-regulation of pro-inflammatory cytokines and chemokines, and in some cases, with an increased expression of anti-inflammatory proteins. In CVD-related diseases, it was demonstrated that genetic inhibition of TREM-1 resulted in the reduction of inflammation and severe AMI in mouse models [69]. Also, TREM-1 inhibition attenuates the inflammation underlying atheroma buildup by decreasing TREM-1 and TLR interaction and downstream signaling [12].

The consideration of TREM-1 reaction pathways for CVD biomarkers is one of the wide venues which studies should endeavor to delve in, since it remains a challenge for healthcare providers to predict cardiovascular events. In fact, atherosclerosis, which is the cause of AMI and stroke, is very difficult to detect by imaging [86]. A discovery of unstable atheroma at the subclinical stage is sure to be life-saving. Angiography is the most accurate and often used method but is very invasive. Echography and MRI cannot yet detect sub-symptomatic plaque. These difficulties call for a simpler and more effective way of finding plaque in blood vessels and early treatment methods. High-sensitivity C-reactive protein levels were suggested earlier, but remains unconvincing due to its non-specificity. As TREM-1 is a key player in the pathogenesis of atherosclerosis, it is worth finding out how it could be used as a biomarker of atherosclerosis susceptibility with at-risk patients. Studies have already shown the trends evidencing that the sTREM-1 level is associated with complications of AMI. sTREM-1 levels were shown to be correlated with the severity of AMI and heart dysfunction during sepsis, but there has not yet been a robust study to prove it being a cause of those aforementioned conditions [15]. We suggest that large-scale and multi-centric investigations of these findings should be undertaken, since they will help to efficiently manage CVD and diminish its mortality.

In the pursuit of finding therapeutic approaches to CVD while utilizing TREM-1, newer TREM-1 inhibitors are being developed. LP-17 is a peptide designed to fit the extracellular Fc component of TREM-1. It is considered to play the role of weakening the dimerization of TREM-1, and competes at the ligand binding site of the receptor [27,87]. LP-17 is a 17 amino acid peptide derived from the extracellular part of TLT-1. LP-17 was shown to down-regulate the expression of TNF-α and IL-1β in monocytes treated with LPS. Another is the triggering receptor expressed on myeloid cells, like how transcript-1 [88,89] and its peptide motif LP-17 were shown to mitigate the dysfunctional downstream signaling of TREM-1. Upon the exposure of human neutrophils and monocytes to LPS, the addition of LP-17 induced lower expression and activity of most pro-inflammatory cytokines and chemokines. Interestingly, LR17 reduced TREM-1 expression in both the initial and later stages of sepsis and decreased the detrimental effects of the chronic inflammation in mice models of sepsis, thus increasing their chances of survival [66,90]. Another dodecapeptide TREM-1 antagonist, LR12 (LQEEDAGEYGCM), was found to be beneficial for atherosclerotic plaque. LR-12 has been shown to block TREM-1 and reduces its activation of monocytes/macrophages to form foam cells in atherosclerosis pathogenesis [12,91]. Furthermore, it has recently been found that the multimerization of TREM-1 which causes its activation is inhibited by LR12 [92]. Also, curcumin extracted from the yellow pigment of turmeric has been shown to inhibit TREM-1 down-streaming signaling. The diferuloylmethane has been shown to modulate inflammation through the inhibition of TREM-1 expression in macrophages and the lungs of septic mice [93]. Endogenous prostaglandin E2 has been found to modulate TREM-1 expression in LPS-rich environments in vitro as well in vivo [94]. Recently, TREM-1 inhibition has been found to benefit the signaling chain homooligomerization (SCHOOL) model that targets the interaction of TREM-1 with DAP-12. In experimental pancreatic or pulmonary cancers, peptides engineered by the SCHOOL technique has been shown to reduce tumor growth and increase survival rates [95]. In fact, the TREM-1 SCHOOL peptide GF9, in free and HDL-bound form, has shown to be an antitumor and to be able to amend sepsis in experimental models [96]. All these advancements in using TREM-1 inhibition as a therapeutic approach will benefit the search for a cure in regard to CVDs.

In dyslipidemic conditions, TREM-1 expression is upregulated in the circulating myeloid cells. In addition to this, TREM-1 also synergizes with high-cholesterol diet (HFCD)-induced monocytosis to promote pro-inflammatory and atherogenic cytokine production and foam-cell formation of macrophages [97]. In a study of renal tissues with diabetic nephropathic rats, vitamin D supplementation has shown to suppress TREM-1 expression, macrophage adhesion, and migration [98]. The effect of vitamin D and its immunomodulatory role in coronary artery atherosclerosis has already been established in various study models. In our previously published study, we have demonstrated the protective nature of vitamin D against atherosclerosis in hypercholesterolemic swine via controlling cholesterol efflux and macrophage polarization through increased CYP27A1 activation [99]. This signals an interesting venue in exploring the influence of epigenetics on the expression of TREM-1 and its actions.

Overall, these findings need to be expanded on further with CVD-specific animal models, through preferably with humanoid models, such as swine. We predict that acute diseases like AMI and endocarditis will be easier to attain, than chronic diseases like atherosclerosis which requires months, or even years to develop.

## 6. Conclusions

**Figure 2:**
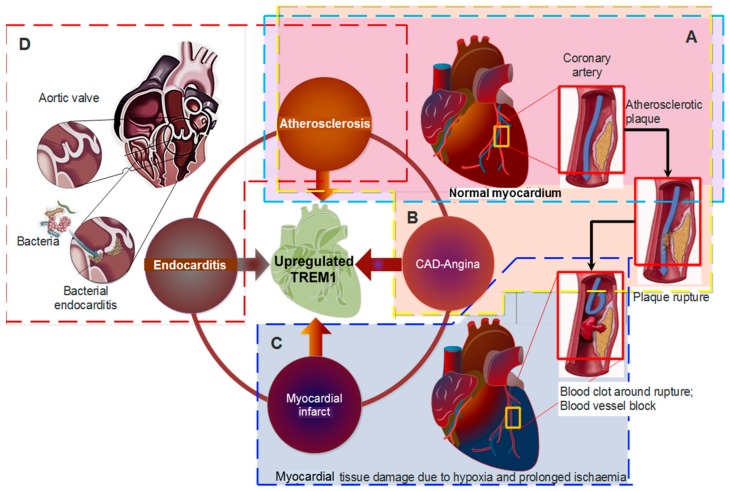
Role of TREM-1 in cardiovascular diseases and potential. (**A**) TREM-1 upregulation is implicated by atheroma formation in the artery and its destabilization. TREM-1 modulation will reduce arthrosclerosis. (**B**) TREM-1 plays a role in atherosclerotic plaque enlargement and instability, which manifests clinically by either coronary artery disease (CAD) or angina, when the coronary is partially clogged or (**C**) AMI when it is completely blocked. TREM-1 inhibition will reduce AMI events in at-risk patients. (**D**) TREM-1 is also the link to the proclivity in developing endocarditis. TREM-1 inhibition can protect at-risk patients.

TREM-1 has an important role as an inflammatory orchestrator and player. It functions by interacting with PRRs and their putative DAMPS and PAMPS. We believed in CVD, PGLYRP-1 alone or linked to PG is the putative ligand of TREM-1 implicated in atherosclerosis. Our insinuation is based on how bacteria were found to play a part in pro-inflammatory reactions leading to atherosclerosis and the LPS component of the bacteria acting as DAMPS to activate TLRs, that are found to interact in synergy with TREM-1. We foresee that any intervention that will downplay the role of TREM-1 will be beneficial in the long run to prevent atherosclerosis and its detrimental consequences of AMI, stroke, or ischemia of the extremities (Figure 2).

Since TREM-1 contributes to the complications following AMI in regard to its immediate and future outcomes, we predict that any modulation of TREM-1 during the management of AMI will be beneficial for the patient. Still, this progress owes to the discovery of the ligand that is involved in the interplay leading to various steps of AMI pathogenesis and complications.

Overall, TREM-1 in CVDs study is at its beginning. The pathogenesis, as well as the therapeutic mechanistic approaches, warrant focus. There are other CVDs that are not yet studied in respect of TREM-1. Because of its role in chronic inflammation (which is the hallmark of CVDs) such as hypertension, valvular diseases, peripheral artery diseases, cardiomyopathy, and heart failure, TREM-1 implications should be investigated for their development and complications, and their treatments should be studied also.

## Figures and Tables

**Table 1:** Involvement of TREM-1 in cardiovascular disease.

CVD	Approach	Outcome	Model Ref
Atherosclerosis	Effect of oxLDL on TREM-1 expression in macrophages during atherogenesis	oxLDL increased TREM-1 expression and its interaction with TLR-4 to amplify inflammation. This action is reduced by TREM-1 silencing or inhibition	M [66]
Atherosclerosis	Role played by TREM-1 in macrophages’ involvement in atherosclerosis	TREM-1, in association with TLR-4, contributes to formation of foam cell derived from macrophages through inflammatory response	M [12]
Atherosclerosis	TERM-1 polymorphism is associated with atherosclerosis severity	rs4711668 polymorphism within TREM-1 gene and TLR-2 are associated coronary atherosclerosis, TLR-1,4,6 with mild coronary atherosclerosis.	H [61]
Atherosclerosis	TREM-1 expression on dendritic cells (DC) in atherosclerotic plaques	TREM-1 was upregulated in DCs of atherosclerotic plaque and was positively correlated with plaque destabilization	H [59]
Atherosclerosis	TREM-1 expression in vulnerability of atheroma plaque	Increased expression of TREM-1 in VSMCs is associated with plaque vulnerability	H [60,67]
Acute myocardial infarction	sTREM-1 level regulation by its polymorphism and plasma sl-selectins level	SNP (rs2234246) is associated with increased plasma sTREM-1 and l-selectin	H [68]
Acute myocardial infarction	Role played by TREM-1 in inflammatory response after AMI	TREM-1 deletion or inhibition decreases inflammation after AMI	M [69]
Acute myocardial infarction	TREM-1 inhibition by LR12’s effect on reperfusion injury after MI	TREM-1 inhibition by LR-12 amends the reperfusion injury of the myocardia	S [70]
Acute myocardial infarction	TREM-1 expression in causing innate immune and inflammatory responses after myocardial infarcts	TREM-1 genetic inhibition reduced inflammation and sTREM-1 level. TREM-1 is positively correlated with AMI severity	M [15]
Coronary artery diseases	TREM-1 and TLR polymorphisms in CAD	Polymorphisms in TREM-1 and in TLRs were robustly associated to CAD	H [61]
In-stent restenosis	Association of sTREM-1 with in-stent restenosis and expression of TREM-1 in VSMCs	sTREM-1 was elevated in patient with stent restenosis, and TREM-1 induces VSMCs inflammation, migration, and proliferation	H [64]
Myocardial dysfunction in septicemia	Association of level of sTREM-1 and severity of myocardial dysfunction in septicemia	sTREM level predicts myocardial dysfunction in septicemia	H [71]
Myocardial dysfunction	Association of TREM-1 with LPS-induced ventricular dysfunction	TREM-1 plays a significant role in LPS-induced ventricular dysfunction	M [72]
Infective Endocarditis	How polymorphism in TREM-1 and TLRs affects the outcome of IE	No association was found between SNPs within TREM-1 genes and the outcome of IE	H [54]
Infective Endocarditis	How heredity of TREM-1 variation could affect the susceptibility and outcome of IE	Only rs1817537 polymorphism is associated with high susceptibility to IE	H [73]
Cardiac transplant	How TREM-1 and antigen-presenting cells affect alloreactive CD4 and lymphocytes	TREM-1 contribute to the differentiation and proliferation of CD-4 positive lymphocytes	M [65]
Cardiac arrest after heart Surgery	sTREM-1 level after cardiac events without infection	TREM-1 along with procalcitonin increase during cardiac events and are not specific to infection but to inflammation	H [7]

H: human; M: mouse; S: swine.
